# Pharmacokinetics of Salicylic Acid Following Intravenous and Oral Administration of Sodium Salicylate in Sheep

**DOI:** 10.3390/ani8070122

**Published:** 2018-07-18

**Authors:** Shashwati Mathurkar, Preet Singh, Kavitha Kongara, Paul Chambers

**Affiliations:** 1 1B, He Awa Crescent, Waikanae 5036, New Zealand; 2 School of Veterinary Sciences, College of Sciences, Massey University, Palmerston North 4474, New Zealand; P.M.Singh@massey.ac.nz (P.S.); K.Kongara@massey.ac.nz (K.K.); J.P.Chambers@massey.ac.nz (P.C.)

**Keywords:** NSAIDs, salicylic acid, sodium salicylate, HPLC, sheep, pharmacokinetics

## Abstract

**Simple Summary:**

Scarcity of non-steroidal anti-inflammatory drugs (NSAID) to minimise the pain in sheep instigated the current study. The aim of this study was to know the pharmacokinetic parameters of salicylic acid in New Zealand sheep after administration of multiple intravenous and oral doses of sodium salicylate (sodium salt of salicylic acid). Results of the study suggest that the half-life of the drug was shorter and clearance was faster after intravenous administration as compared to that of the oral administration. The minimum effective concentration required to produce analgesia in humans (16.8 µL) was achieved in sheep for about 0.17 h in the current study after intravenous administration of 100 and 200 mg/kg body weight of sodium salicylate. However, oral administration of these doses failed to achieve the minimum effective concentration as mentioned above. This study is of significance as it adds valuable information on pharmacokinetics and its variation due to breed, species, age, gender and environmental conditions. As per the authors’ knowledge, this is the only study showing detailed information about absorption, distribution and elimination of salicylic acid in New Zealand Sheep. An intravenous administration of sodium salicylate at 100 and 200 mg/kg dose may produce analgesia in sheep, which requires further investigation using pharmacokinetic–pharmacodynamic (PKPD) integration or modelling techniques.

**Abstract:**

The pharmacokinetics of salicylic acid (SA) in sheep was evaluated following intravenous (IV) and oral administration of sodium salicylate (sodium salt of salicylic acid) at different doses. Six healthy sheep were administered sodium salicylate (SS) IV at doses of 10, 50, 100 and 200 mg/kg body weight and another six sheep were drenched with 100 and 200 mg/kg of SS orally. Both studies were randomised crossover trials. A one-week washout period between each treatment was allowed in both studies. Blood samples were collected at 0, 15, 30 min and 1, 2, 4 and 6 h after IV and oral SS administrations. Plasma SA concentrations were determined using high-performance liquid chromatography (HPLC) with diode array detection method. Pharmacokinetic variables were calculated in a non-compartmental model. The elimination half-life (T_1/2 el_) of SA after IV administration of 200 mg/kg SS was 1.16 ± 0.32 h. Mean bioavailability of SA was 64%, and mean T_1/2 el_ was 1.90 ± 0.35 h, after 200 mg/kg of oral SS. The minimum plasma SA concentration (16.8 µg/mL) reported to produce analgesia in humans was achieved after IV administration of 100 and 200 mg/kg SS in sheep for about 0.17 h in this study. Experiments on pharmacokinetic–pharmacodynamics modelling are required to determine the actual effective plasma concentration range of SA in sheep.

## 1. Introduction

Sodium salicylate (SS) is a non-steroidal anti-inflammatory drug (NSAID) with anti-pyretic, analgesic and anti-inflammatory properties. It has been used as a pro-drug of salicylic acid (SA), which is the active anti-inflammatory agent. The use of SS as a NSAID in humans is well established [1,2]. In animals such as cattle, intravenous SS (50 mg/kg) attenuated the plasma cortisol responses associated with castration in calves [3,4]. In sheep, no reports are available on use of SS for providing analgesia and its anti-inflammatory effects. The pharmacokinetic variables of salicylates are not consistent in animals as they are influenced by the age, gender, weight and breed of the animal [5,6].

Sulaiman and Kumar [7] reported the pharmacokinetics of SS after a single intravenous (IV) and oral dose of SS (100 mg/kg) in Bannur (local Indian breed) sheep. Another pharmacokinetic study of salicylates in the desert sheep was reported by Ali [8]. In this study, they analyzed the total plasma salicylate concentrations and calculated the pharmacokinetic parameters after IV and intramuscular administration of DL-lysine-acetyl salicylate (20 mg/kg). In both studies, total salicylates were analyzed as against salicylic acid. No other studies have been reported on the pharmacokinetics of salicylates in the sheep.

The pharmacokinetics of acetyl salicylate or aspirin which is another pro-drug of salicylic acid have also been conducted in cattle, horse, rabbit, goat, camel, cat and avian species. In cattle, aspirin was commonly used to treat respiratory ailments caused by bacteria and viruses [9]. The pharmacokinetics of aspirin was studied by Whittem et al. (1996) after administration of DL-lysil-acetyl salicylate IV in cattle at a dose rate of 26 mg/kg body weight [10]. The elimination half-life of salicylate reported in this study was 30 min and volume of distribution was 1.2 L/kg. Coetzee et al. (2007) [4] studied the pharmacokinetics of SS in calves at 50 mg/kg body weight and obtained similar results as Whittem and co-workers. Another pharmacokinetic study in cattle conducted by Short et al. (1990) [11] reported that 54% of SS is excreted in its original form after IV administration, while less than 12% of SS was eliminated through urine after its oral administration. Salicyluric acid, (the glycine conjugate) was reported to be a major metabolite of SS excreted by cattle after its IV and oral administration. In goats, when SS was administered IV and orally, its recovery as SS in urine was 67.9% and 30.2%, respectively, and salicyluric acid was also excreted as a major metabolite [12]. Salicyluric acid, (the glycine conjugate) was reported to be a major metabolite of SS excreted by cattle after its IV and oral administration. In humans, the major metabolite of salicylic acid eliminated in urine was also salicyluric acid [12]. The pharmacokinetic studies in cattle and goats could be of relevance to compare with sheep, as all these species are ruminants. However, inter-species pharmacokinetic differences should always be considered.

Inter-species variation in pharmacokinetics of salicylates is evident from the studies reported by various authors [4,7,13]. A wide disparity of half-life (about 26 min in goats to 22 h in cats), and clearance (ranging from 0.04 L/h/kg in rabbits to 5.31 L/h/kg in cats) in different animal species can be observed. Plasma protein binding, pH of the urine, bioavailability, and extent of absorption from the site of administration could be the factors contributing towards the interspecies variation in the pharmacokinetics of salicylates [12,14]. Also, intra-species and inter-individual variation has been observed by some researchers in cattle [9,10]. Consequently, a distinct pharmacokinetic study in each species is warranted.

The aim of the present study was to determine the pharmacokinetics of salicylic acid at the different dose rates of SS administered IV and orally in sheep.

## 2. Materials and Methods

### 2.1. Reagents and Drugs

Acetonitrile HPLC grade was purchased from Merck, KGaA; 64271 Darmstadt, Germany, and Orthophosphoric acid from BDH Limited, Poole, England. SS (Laboratory grade reagent) was purchased from Fisher Scientific UK. The stock solution of SS was prepared in Milli Q water (Milli-q PFplus system, Millipore Cooperation, Burlington, MA, USA) by dissolving 0.1 g SS in 100 mL. The working standard solutions were prepared in mobile phase fresh daily from the stock solution. Six different concentrations of SS standards, 50 µg/mL, 5 µg/mL, 0.5 µg/mL, 0.25 µg/mL, 0.125 µg/mL and 0.0625 µg/mL were prepared from stock solution in mobile phase (diluting the stock solution with mobile phase to get desired standard concentration) to make a calibration curve.

SS oral and injection solution for administration was made by dissolving 50 g of SS (Laboratory grade reagent, Fisher Scientific, Loughborough, UK) in 100 mL of Milli-Q water. SS solution was filtered through a syringe filter (0.45 μm, Phenomenex Inc., Auckland, New Zealand) prior to administration.

### 2.2. Animals and Experimental Procedure

Sheep were sourced from the herd maintained for teaching and research by the Large Animal Teaching Unit (LATU), School of Veterinary Science, Massey University, Palmerston North. Sheep were kept under typical husbandry conditions with free access to grass grazing and water. The study animals were clinically examined before enrolment in this study and at least once a day during the study. Any sheep showing the signs of lameness, foot rot, inflammation or pain were excluded from the study. This study was approved by the Massey University Animal Ethics Committee (protocol 13/18).

#### 2.2.1. Intravenous Pharmacokinetic Study

Six healthy Romney cross sheep (2 males and 4 females), age 6 months with mean weight 42.25 ± 5.7 kg (mean ± SD), were used in a randomised cross-over design. Sheep were manually restrained and clipped over the left and right jugular veins. An 18 gauge 2 inch catheter was placed in a jugular vein aseptically for blood collection, while the contralateral vein was used for injection of the drug at four different dose rates; 10, 50, 100 and 200 mg/kg with a one-week wash out period between each treatment.

#### 2.2.2. Oral Pharmacokinetic Study

Six healthy Romney cross sheep (3 males and 3 females), age 8 months with mean body weight 38.66 ± 2.7 kg (mean ± SD), were used in a randomised cross-over design. Sheep were manually restrained and clipped over either right or left jugular vein. An 18 gauge 2 inch catheter was placed in a jugular vein for blood collection. All sheep were drenched with 100 and 200 mg/kg SS solution with a one-week washout period between the two treatments.

### 2.3. Sample Collection

A 3 mL blood sample was collected in a heparinised vacutainer (BD Vacutainer Green (LH, 10 mL, Phoenix Pharm, Mairangi Bay, Auckland, New Zealand) at 0, 15, 30 min and 1, 2, 4, 6 h after IV and oral administrations of SS with each dose rate.

### 2.4. Sample Preparation

All samples and spiked plasma standards were analysed by using solid phase extraction (SPE) method using Phenomenex strata-X 3 mL, 60 mg SPE cartridge (Phenomenex, Auckland, New Zealand). A 500 µL of plasma was spiked with either 500 µL of a known concentration of SS standard or Milli-Q water (in case of plasma sample obtained from the test sheep administered with SS). The sample was vortex-mixed for 2 min. The cartridge was activated by 1 mL (100%) methanol followed by equilibration with 1 mL of Milli-Q water. A 1 mL sample was loaded followed by a single wash with 20% methanol. The cartridge was then dried for 10 min under vacuum. The elution was carried out with 1 mL of 100% methanol, collected in a glass test tube. This eluent was dried under the gentle stream of compressed air at 40 °C and reconstituted with 300 µL of mobile phase. The sample was vortex-mixed for 30 s and then centrifuged at 14,000 *g* for 10 min. A 50 µL sample volume was injected twice into the HPLC system.

### 2.5. Sample Analysis

The analysis was carried out using High-Performance Liquid Chromatography with Diode detector. This system consisted of LC-20 AD pumps, SIL-20 AC HT auto-injector, SPD-M20A diode array detector, CTO-20A column oven, DGU-20 A3 degasser (Shimadzu, Kyoto, Japan). Sodium salicylate was separated with Synergi Hydro^®^ (C18, RP 4µ, 80 Å, LC Column 150 × 4.6 mm) column (Phenomenex, Auckland, New Zealand) at 40 °C. The mobile phase consisted of Milli-Q water (71%), acetonitrile (28%) and orthophosphoric acid (1%), pH 2.54. Mobile phase was filtered through 0.2 µm membrane filter. Analysis of the samples was carried out under isocratic conditions at 0.8 mL/minute flow rate. All the chromatograms were analysed at 230 nm wavelength. All the chromatograms were analysed for peak height, area and concentration for the unknowns using LC solutions software (Shimadzu, Kyoto, Japan).

### 2.6. Validation Protocol

The HPLC using Diode Array Detector (DAD) method and the sample preparation method were validated by following a standard validation procedure [15]. Blank plasma was spiked with the standards of SS to acquire the standard curve and the recovery of the drug was calculated by comparing the set of standards in mobile phase with the set of standards spiked in the blank plasma. Linearity were determined by analysing 500 μL of sheep plasma spiked with five different concentrations of SS ranging from 0.125 to 50 μg/mL. The data for peak area thus obtained were analysed by linear regression using Prism 6 for Macintosh (GraphPad Software, Inc., San Diego, CA, USA). Intra- and inter-day variation was calculated for all the five concentrations spiked with blank plasma analysed for three consecutive days. The samples for each concentration were prepared every day and were run in three different batches each day in triplicate for intra-day variation. This was repeated for three different days to check inter-day variation. The lower limit of detection of SS was set at signal-to-noise ratio of 3:1. The selectivity of the method was determined by analysing the drug-free plasma sample from 10 different sheep, processed following the same sample preparation method.

### 2.7. Pharmacokinetic Analysis

The pharmacokinetic parameters were calculated in a non-compartmental model using the standard equations in an Excel spreadsheet (Microsoft, Redmond, WA, USA). These parameters included half-life of terminal elimination phase (T_1/2__ƛz_), area under the curve extrapolated from time zero to infinity (AUC_0–∞_), area under the moment curve extrapolated from time zero to infinity (AUMC_0–∞_), Volume of distribution (V_d_, mL/kg), clearance (Cl, mL/min/kg), bioavailability (F, %) and mean resident time (MRT, min).

### 2.8. Statistical Analysis

The Kolmogorov–Smirnov test was performed to check the normality of the data obtained from both intravenous and oral pharmacokinetic analyses. Normally distributed data for intravenous pharmacokinetics of SS were then analysed by one-way ANOVA (analysis of variance) with post hoc Tukey’s multiple comparison test. Non-normally distributed data were analysed using Kruskal–Wallis test with Dunn’s multiple comparison test as a post hoc test. Normally distributed data for oral pharmacokinetics of SS were analysed for significant differences between the pharmacokinetic parameters of the two dose rates (oral 100 and 200 mg/kg SS) using a paired *t* test. Non-normally distributed data were analysed using Mann–Whitney U test. All the statistical analyses were carried out using Prism 6 for Macintosh (GraphPad Software, Inc., San Diego, CA, USA) and *p* < 0.05 were considered significant. The data are reported in mean ± standard deviation.

## 3. Results

### 3.1. HPLC Method Validation

**Figure 1:**
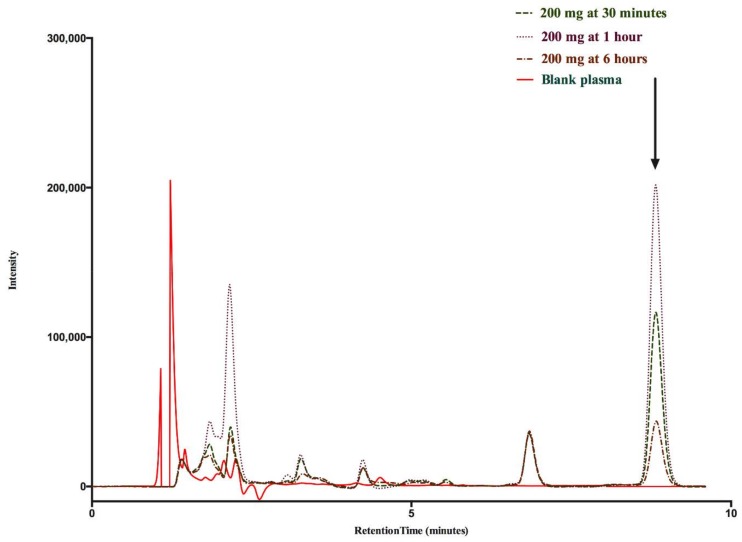
Chromatographs showing SA peaks at 30 min, 1 h (T_max_) and 6 h after oral administration of 200 mg/kg SS in sheep; while a drug-free/blank plasma of a sheep represented by orange line has no peak at the retention time of salicylic acid. Arrow represents salicylic acid peak.

The chromatograms showing peaks of SA at different concentrations are shown in Figure 1. Drug recovery varied from 97 to 102%. Overall relative standard deviation (RSD) (mean ± SD) for recovery was 1.32 ± 0.39%. The RSD for intra-day and inter-day variability (mean ± SD) were 1.45 ± 1.03 and 1.58 ± 1.26%, respectively. Linearity of the method for SA was 0.9996. The limits of detection (LOD) of SS was 62.5 ng (0.0625 µg).

### 3.2. Pharmacokinetics of SA after Intravenous Administration of SS in Sheep

**Figure 2:**
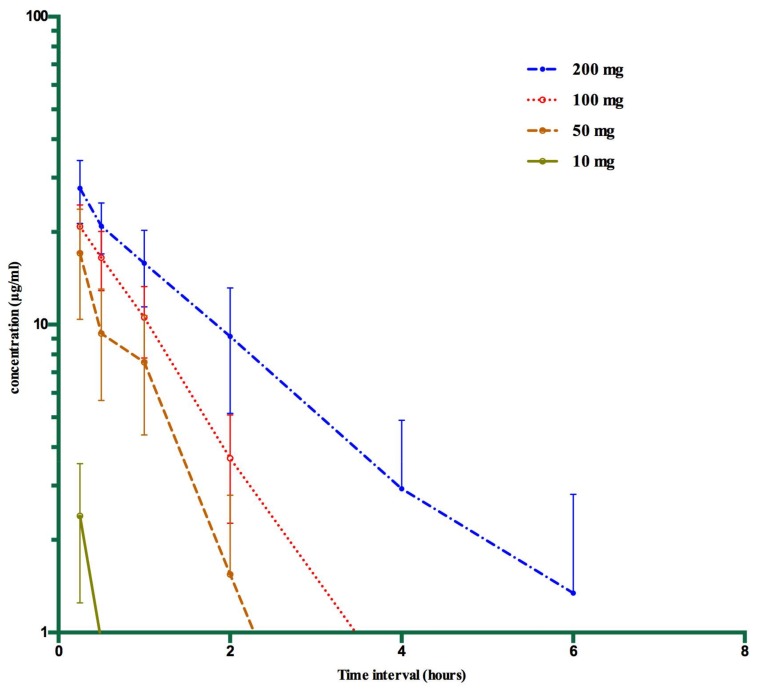
Semi-log concentration–time curve for salicylic acid after all treatments (10, 50, 100, 200 mg/kg) of IV SS in six sheep (mean ± SD).

Pharmacokinetics of SA after IV administration of SS in sheep at different dose rates are given in Table 1. The semi-log concentration–time curve for all treatments (10, 50, 100, 200 mg/kg) of IV SS in sheep is shown in Figure 2. The maximum plasma SA concentration (C_max_) was 2.39 ± 1.14, 17.05 ± 6.65, 20.82 ± 3.64 and 27.72 ± 6.43 µg/mL after 10, 50, 100 and 200 mg/kg of SS, respectively. The lowest concentrations of SA ranged from 0 to 3.74 µg/mL after 200 and 100 mg/kg IV dose and were detected at six hours. At 50 and 10 mg/kg IV dose, SA was detected only till four hours up to 0.03 to 0.4 µg/mL. Sheep administered 100 and 200 mg/kg of SS had overall significantly higher (*p* < 0.05) AUC (area under concentration–time curve) than 50 and 10 mg/kg body weight. Total plasma clearance was significantly higher in sheep administered with 10 mg/kg as compared to higher intravenous doses. The elimination half-life and mean resident time were significantly higher in 200 mg/kg intravenous-dosed sheep as compared to other doses.

### 3.3. Pharmacokinetics of SA after Oral Administration of SS in Sheep

**Figure 3:**
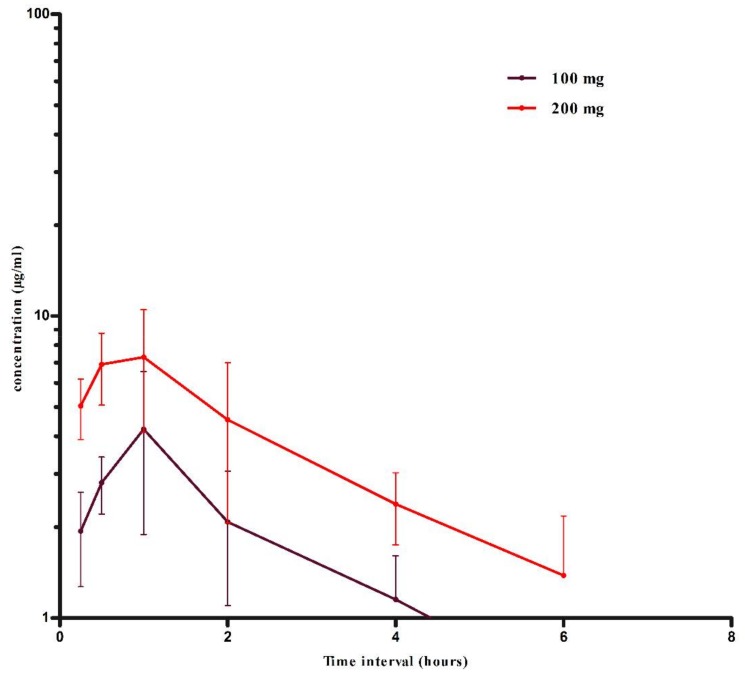
Semi-log concentration–time curve for salicylic acid after 100 and 200 mg/kg treatments of oral SS in six sheep (mean ± SD).

The semi-log concentration–time curve for 100 and 200 mg/kg oral SS in sheep is shown in Figure 3. The non-compartmental pharmacokinetics of sheep with statistical analysis after oral administration of SS at 100 and 200 mg/kg is shown in Table 2. None of the pharmacokinetic parameters were significantly different for oral dose rates 100 and 200 mg/kg except AUMC. Also, high volumes of distribution were observed as compared to the IV study. The C_max_ after a single oral dose for 100 and 200 mg/kg was 4.22 ± 2.33 and 8.27 ± 2.38 µg/mL, respectively. Bioavailability (absolute) of salicylic acid was calculated by using the standard formula [16,17] after comparing with the AUC’s of IV bolus of the SS. However, the animals used in the IV and oral pharmacokinetics studies were different. Therefore, overall average bioavailability for respective doses was used for each animal for further pharmacokinetics analysis. Bioavailability for 200 mg/kg oral SS was 0.64 (64%) and for 100 mg/kg it was 0.53 (53%). 

## 4. Discussion

The objective of this study was to determine pharmacokinetics of salicylic acid after IV and oral administration of SS in New Zealand sheep. Significant differences between the pharmacokinetic parameters were observed at different dose rates of the single IV bolus. The faster clearance at lower dose (10 mg/kg) indicates the dose-dependent elimination of SS. Sodium salicylate follows first-order kinetics in humans at lower doses, while at higher doses a dose-dependent kinetics is observed with respect to elimination [18,19]. When SS metabolises to salicylic acid, it conjugates with glycine to form salicyluric acid, while conjugation with glucuronides results in salicyl phenolic glucuronide and acyl salicyl glucuronide in humans [20]. It is also hydrolyzed to form gentisic acid. When lower doses are administered, salicylic acid forms these metabolites at a faster rate, while at higher doses, it reaches saturation, especially during conjugation with glucuronide [12,21]. Therefore, reduced metabolism results in the accumulation of drug in the plasma, thus increasing the elimination half-life with reduced clearance. Similar variation in clearance was observed with the single IV bolus of anti-neoplastic agent thiotepa (TT) in children and a difference in clearance was observed at different doses of TT [22].

The MRT after oral administration of SS was higher as compared to IV dose due to slower absorption. Half-lives after both oral and IV administration of SS were not significantly different when respective dose rates of both routes are compared. Hence, rapid elimination of SS is evident after oral administration. This shows the necessity of frequent dosing after oral administration to achieve the minimum effective plasma concentration (MEC) of salicylic acid to produce analgesia, which is reported to be 16.8 µg/mL [23] in humans. In the present study, plasma salicylic acid concentration was maintained above the MEC levels for 30 and 15 min after an IV dose of SS at 200 and 100 mg/kg, respectively. However, after IV doses of 50 and 10 mg/kg or a single oral dose of 200 and 100 mg/kg SS could not achieve plasma concentrations above the MEC. Thus, these dose rates may not be effective. In the current study, the plasma salicylic acid concentration to produce analgesia in cattle (25 to 30 µg/mL) [4,9,24] was achieved only for 15 min after 200 mg/kg IV dose of SS. The plasma concentrations of salicylic acid achieved during the oral pharmacokinetic study were similar to the study conducted by Maalouf et al. (2009) in humans, where 162 mg aspirin was administered orally and the plasma salicylic acid concentrations were not higher than 10 µg/mL [25]. However, the species extrapolation of pharmacokinetic parameters is not appropriate [5,26]. The pharmacodynamics (PD) study and subsequent pharmacokinetic pharmacodynamic (PKPD) modelling is required to associate the analgesic efficacy of SS in sheep.

The T_max_ after oral administration of 100 mg/kg SS dose was similar as reported by Sulaiman and Kumar [7] in their study. However, after 200 mg/kg SS oral dose, T_max_ ranged from 0.5 to 1 h within the six sheep in the current study. This shows that individual variation impacts the pharmacokinetic data. The plasma clearance and volume of distribution was higher than Sulaiman and Kumar’s study after both IV and oral administration of SS. A higher volume of distribution could be due to higher absorption of weak acids such as NSAIDs in reticulo-rumen (pH 5.5–6.5) [27,28,29]. Thus, higher distribution after oral administration is explicable. Salicylates have low volumes of distribution ranging from 0.1 to 0.2 L/kg due to the high plasma protein binding, resulting in lower concentrations of free drug in the plasma [30]. Salicylates are also reported to be highly lipophilic [30] due to their weakly acidic nature. This may result in higher distribution in tissues, extracellular fluids and other body fluids that could contribute to the high volumes of distribution as observed in the present study [31,32]. A higher V_d_ could also be due to a higher body fat of animals in the current study as compared to the study conducted by Sulaiman and Kumar with leaner sheep. Similarly, protein deficiency may also affect the pharmacokinetic parameters [33]. However, the protein parameters were not considered in either study. Differences in the pharmacokinetics parameters could also be due to the method of pharmacokinetic analysis and calculations, age, breed and weights of the animals, analytical methods used to determine salicylic acid or salicylate concentration. In the present study, free/unbound plasma salicylic acid concentrations were determined, while in the other study, total serum salicylate concentrations were determined. Also, environmental differences might contribute to the varying pharmacokinetic parameters [34]. It would be valuable to measure the total proteins and lipid profile as well as liver enzymes in plasma.

High inter-animal variation in pharmacokinetic parameters of the present studies supports the wide diversity of pharmacokinetics of SS (Table 3) as described by Levy [26], Riviere and Papich [5] and Bope and Kellerman [35]. Therefore, to extrapolate these parameters from other species to sheep or even from different breeds of the same species is not appropriate.

## 5. Conclusions

In conclusion, an IV dose of 200 mg/kg SS in sheep achieved the MEC of salicylic acid for analgesia in cattle (above 25 µg/mL). Also, MEC of salicylic acid for analgesia in human (16.8 µg/mL) was achieved by two IV dose rates, 100 and 200 mg/kg of SS in sheep. Oral administration of both 100 and 200 mg/kg SS failed to achieve the MEC of salicylic acid for analgesia reported for cattle as well as humans. The current study has significance as no other pharmacokinetic study with sodium salicylate in New Zealand sheep has been conducted. Also, the differences in pharmacokinetic parameters with respect to breeds (different breeds) of the same species (sheep) have been compared and discovered. PKPD modeling or pharmacodynamics experiments are required to determine the actual effective plasma concentration range of salicylic acid in sheep.

## Figures and Tables

**Table 1:** Average non-compartment pharmacokinetic parameters of salicylic acid for all dose treatments (single IV SS bolus in sheep) (mean ± SD).

Parameters	Units	200 mg	100 mg	50 mg	10 mg
C_max_	µg/mL	27.72 ± 6.43 ^a^	20.82 ± 3.64 ^a^	17.05 ± 6.65 ^b^	2.39 ± 1.14 ^c^
AUC_0–∞_	µg·h/mL	47.11 ± 13.02 ^a^	25.95 ± 5.05 ^ac^	14.94 ± 5.39 ^bc^	1.42 ± 1.00 ^b^
AUMC_0–∞_	µg·h^2^/mL	82.50 ± 39.94 ^a^	28.62 ± 10.14 ^ac^	12.32 ± 6.48 ^bc^	1.29 ± 1.98 ^b^
MRT	Hours	1.67 ± 0.47 ^a^	1.07 ± 0.20 ^b^	0.79 ± 0.20 ^b^	0.66 ± 0.47 ^b^
F		1.00 ± 0.00	1.00 ± 0.00	1.00 ± 0.00	1.00 ± 0.00
Cl	L/h/kg	4.52 ± 1.22 ^a^	3.99 ± 0.83 ^a^	3.89 ± 1.93 ^a^	9.29 ± 4.64 ^b^
V_d_	L/kg	7.19 ± 1.12 ^a^	4.16 ± 0.32 ^b^	2.86 ± 0.98 ^b^	5.07 ± 2.23 ^b^
T_1/2_	Hours	1.16 ± 0.32 ^a^	0.74 ± 0.14 ^b^	0.54 ± 0.14 ^b^	0.46 ± 0.32 ^b^

Differences are considered significant when *p* < 0.5, superscripts represent significant difference. C_max_, Maximum plasma concentration; AUC_0–∞_, Area under the curve from zero to infinity; AUMC_0–∞_, Area under the moment curve from zero to infinity; MRT, Mean resident time; F, Bioavailablity; Cl, Clearance; V_d_, Volume of distribution; T_1/2_ Terminal half life.

**Table 2:** Average of all non-compartment pharmacokinetic parameters of salicylic acid after single oral doses of SS in sheep (mean ± SD).

Parameters	Units	200 mg	100 mg
AUC_0–∞_	µg·h/mL	24.45 ± 7.57	12.26 ± 3.39
AUMC_0–∞_	µg·h^2^/mL	65.16 ± 16.57 ^a^	32.70 ± 8.19 ^b^
C_max_	µg/mL	8.27 ± 2.38	4.22 ± 2.33
MRT	Hours	2.75 ± 0.51	2.69 ± 0.15
F		0.64 ± 0.00	0.53 ± 0.00
Cl	L/h/kg	5.79 ± 2.16	4.62 ± 1.38
V_ss_	L/kg	16.45 ± 8.33	12.48 ± 4.02
T_1/2_	Hours	1.90 ± 0.35	1.86 ± 0.11
MAT	Hours	1.07 ± 0.66	1.61 ± 0.26
K_a_	1/h	0.64 ± 0.17	0.63 ± 0.11

Differences are considered significant when *p* < 0.05.

**Table 3:** Pharmacokinetic parameters of SS in different animal species after IV administration.

Species	Dose (mg/kg)	Form	V_d_ (L/kg)	Cl (L/h/kg)	T_1/2_ (h)	Workers
Sheep	100	SS	4.16	3.99	0.74	Current study
Sheep	50	SS	2.86	3.89	0.54	Current study
Sheep	100	SS	0.342	0.26	0.56	Sulaiman & Kumar [7]
Goats	44	SS	0.129	0.15	0.80	Davis & Westfall (1972) [13]
Calves	50	SS	0.24	0.16	1.23	Coetzee et al. (2007) [4]
